# New Insights into the Classification and Integration Specificity of *Streptococcus* Integrative Conjugative Elements through Extensive Genome Exploration

**DOI:** 10.3389/fmicb.2015.01483

**Published:** 2016-01-06

**Authors:** Chloé Ambroset, Charles Coluzzi, Gérard Guédon, Marie-Dominique Devignes, Valentin Loux, Thomas Lacroix, Sophie Payot, Nathalie Leblond-Bourget

**Affiliations:** ^1^DynAMic, Faculté des Sciences et Technologies, Université de Lorraine, UMR 1128Vandœuvre-lès-Nancy, France; ^2^DynAMic, Institut National de la Recherche Agronomique, UMR 1128Vandœuvre-lès-Nancy, France; ^3^Laboratoire Lorrain de Recherche en Informatique et ses Applications, Faculté des Sciences et Technologies, Université de Lorraine, UMR 7503Vandœuvre-lès-Nancy, France; ^4^CNRS, Laboratoire Lorrain de Recherche en Informatique et ses Applications, UMR 7503Vandśuvre-lès-Nancy, France; ^5^UR 1404 Mathématiques et Informatique Appliquées du Génome à l’Environnement, Institut National de la Recherche AgronomiqueJouy-en-Josas, France

**Keywords:** integrative and conjugative elements, T4SS, integrase, integration site, *Streptococcus*

## Abstract

Recent genome analyses suggest that integrative and conjugative elements (ICEs) are widespread in bacterial genomes and therefore play an essential role in horizontal transfer. However, only a few of these elements are precisely characterized and correctly delineated within sequenced bacterial genomes. Even though previous analysis showed the presence of ICEs in some species of *Streptococci*, the global prevalence and diversity of ICEs was not analyzed in this genus. In this study, we searched for ICEs in the completely sequenced genomes of 124 strains belonging to 27 streptococcal species. These exhaustive analyses revealed 105 putative ICEs and 26 slightly decayed elements whose limits were assessed and whose insertion site was identified. These ICEs were grouped in seven distinct unrelated or distantly related families, according to their conjugation modules. Integration of these streptococcal ICEs is catalyzed either by a site-specific tyrosine integrase, a low-specificity tyrosine integrase, a site-specific single serine integrase, a triplet of site-specific serine integrases or a DDE transposase. Analysis of their integration site led to the detection of 18 target-genes for streptococcal ICE insertion including eight that had not been identified previously (*ftsK*, *guaA*, *lysS*, *mutT*, *rpmG*, *rpsI*, *traG*, and *ebfC*). It also suggests that all specificities have evolved to minimize the impact of the insertion on the host. This overall analysis of streptococcal ICEs emphasizes their prevalence and diversity and demonstrates that exchanges or acquisitions of conjugation and recombination modules are frequent.

## Introduction

Streptococci are Gram positive bacteria belonging to the phylum of Firmicutes. This genus comprises 110 recognized species (^1^July 24, 2015). Almost all streptococci are commensal or pathogen of humans and/or animals. Numerous streptococci, such as *Streptococcus pneumoniae*, *Streptococcus pyogenes*, *Streptococcus mutans* or *Streptococcus agalactiae*, are responsible for a wide variety of diseases worldwide, ranging from mild to invasive infections that have a severe impact on human and animal health and lead to significant morbidity and mortality (Mitchell, 2003; Kohler, 2007). Streptococci are also ubiquitously present as commensal inhabitants of the gastro-intestinal tracts of healthy adults and/or newborns. *Streptococcus* *salivarius*, in particular, is one of the first colonizers of the human oral cavity (Park et al., 2005; Nakajima et al., 2013) and is also a dominant part of the early life human intestinal microbiota (Arrieta et al., 2014). At last, two species deriving from commensal streptococci, *S. thermophilus* and *S. macedonicus* are used as starters in dairy industry to transform milk in yogurt and/or cheese (Franciosi et al., 2009).

During the last 20 years, it has become increasingly apparent that horizontal gene transfer (HGT) of genomic islands plays a key role in bacterial evolution and adaptation (Hacker and Kaper, 2000; Hacker and Carniel, 2001; Dobrindt et al., 2004; Juhas et al., 2009). In essence, genomic islands are chromosomal segments acquired by HGT that carry gene sets enhancing the fitness of their hosts. Recent data suggest that numerous genomic islands correspond to non-canonical classes of mobile genetic elements (MGEs) that can transfer by conjugation or are non-mobile elements deriving from such MGEs (Bellanger et al., 2014). Among them, the integrative and conjugative elements (ICEs) are mobile elements integrated in bacterial chromosomes or plasmids which encode their own excision, their transfer by conjugation, and their integration (Burrus et al., 2002b; Bellanger et al., 2014).

Integration of ICEs is catalyzed by three phylogenetically and structurally unrelated families of enzymes: tyrosine integrases, serine integrases, and DDE transposases (Wozniak and Waldor, 2010; Bellanger et al., 2014). Both tyrosine and serine integrases usually catalyze site-specific recombination between small (2–60 bp) similar or identical sequences included in the *attI* site of the circular form of the ICE and the *attB* site of the bacterial genome. This leads to the formation of *attL* and *attR* sites flanking the integrated elements; as a consequence, the integrated ICE is flanked by direct repeats (DR). Usually, tyrosine integrases catalyze ICE integration in a large array of specific sites, including the 3′ end of tRNA genes and the 3′ or 5′ end of genes encoding various housekeeping proteins (Bellanger et al., 2014). One exception is the tyrosine integrase of Tn*916* that shows low integration specificity; as a consequence, Tn*916* and its relatives are not flanked by DRs. Knowledge of the integration specificity of serine integrases from ICE is scarce. The third family of enzymes, DDE transposases, catalyzes transposition of DNA segments. Binding of the enzyme to terminal inverted repeats (IRs) at the extremities of elements enables strand cleavage required for the transposition reaction. DDE transposases have a low specificity of integration and catalyze the duplication of the target sequence (2–13 bp). Up to now, only one subfamily of DDE transposases was described for streptococcal ICEs. These DDE integrases catalyze the integration 15–16 bp upstream of the -35 box of the promoter region of various genes (Brochet et al., 2009; Guérillot et al., 2013).

The first step of the conjugative transfer is the excision of the ICE as a circular form that is ensured by the same enzyme as for integration. In general, tyrosine integrases need additional co-factors to carry out the reverse excision reaction (Groth and Calos, 2004); these are encoded by the element. So far, most ICEs (including all ICEs from Firmicutes) transfer as single-strand DNA. The transfer of the excised ICE would be similar to the transfer of conjugative plasmids that is well-known in Gram negative bacteria (Smillie et al., 2010; Low et al., 2014). The circularized ICE DNA is taken over by a relaxosome, a complex that includes a relaxase. A relaxase is a *trans*-esterase, acting as a dimer, that catalyzes a site and strand-specific cleavage at the *nic* site of the origin of transfer (*oriT*) of its cognate ICE. The relaxase, covalently bound to the 5′ end of the single-stranded DNA, is then recognized by the membrane-associated coupling protein (CP) that interacts with a type IV secretion system (T4SS). T4SSs are ATP-powered and multi-protein complexes that span the cellular envelopes of the donor cell in Gram negative bacteria. CP and T4SS translocate the DNA-relaxase complex through membranes and cell walls into the recipient cell. A rolling-circle replication of the element is likely concomitant to its transfer so that the ICE is not lost in the donor cell. Finally, the relaxase achieves the transfer by recircularizing the ICE (for a review see (Bellanger et al., 2014)). Although the conjugative transfer of DNA in Firmicutes is poorly known, it relies on relaxase, CP and T4SS (Goessweiner-Mohr et al., 2013; Guglielmini et al., 2014). Recent analyses suggest that T4SSs of Firmicutes include an homolog or analog of most T4SS membrane-spanning proteins found in inner membranes of Gram negative bacteria including the ATPase VirB4, VirB3, VirB6, VirB8 and the cell-wall degrading enzyme VirB1 (Goessweiner-Mohr et al., 2013; Guglielmini et al., 2014; Leonetti et al., 2015).

Like all other bacterial MGEs (Toussaint and Merlin, 2002), ICEs have a modular structure, i.e., the genes involved in the same biological function (such as conjugation or integration/excision) are physically linked. In addition to the integrase and recombination directionality factor genes, the integration/excision module includes the recombination site *attI*. It is thus generally located at one end of the integrated element. The conjugation module includes genes coding for the T4SS, the CP, the relaxase (and eventually accessory proteins of the relaxase) and *oriT*. The regulation module encodes all the genes involved in the regulation of ICE dissemination and maintenance. In addition to modules dedicated to gene transfer, all ICEs also carry at least one adaptation module that encodes adaptive traits that might be beneficial for bacteria under certain growth or environmental conditions. Adaptation modules are highly variable and include genes involved in antimicrobial resistance, virulence or alternative metabolic pathways (Burrus et al., 2002a,b; van der Meer and Sentchilo, 2003; Schubert et al., 2004; Heather et al., 2008; Croucher et al., 2009; Chuzeville et al., 2012).

Although ICEs have a major impact on gene flow and genome dynamics in bacteria, their prevalence and diversity remain largely underscored (Bellanger et al., 2014).

In this work, we took advantage of the increase of publicly available genomic sequences (124 complete genomes of *Streptococcus* available at the beginning of this work) to search for ICEs using the combined presence of signature proteins (from conjugation and integration/excision modules). Coding sequences (CDSs) encoding these signature proteins were localized on the chromosomes and a strategy was developed to search for ICEs boundaries and to identify their integration site. This work (i) gives a general overview of the high prevalence and diversity of ICEs within *Streptococcus* species, (ii) identifies their numerous sites of insertion, and (iii) sheds light on their phylogenetic relationships and on their modular evolution.

## Materials and Methods

### Genomes Examined and Database of Reference Proteins

The dataset of the 124 complete chromosomes from *Streptococcus* species available at the beginning of this work was taken from GenBank (^2^last accessed December 2013).

The initial database of reference proteins contains signature proteins from ICEs reported for Firmicutes in the literature at the beginning of this study. It includes protein sequences from 50 tyrosine integrases, 13 serine integrases, two DDE transposases, 50 relaxases, 37 CP, and 26 VirB4 proteins.

### Search Strategy

**FIGURE 1:**
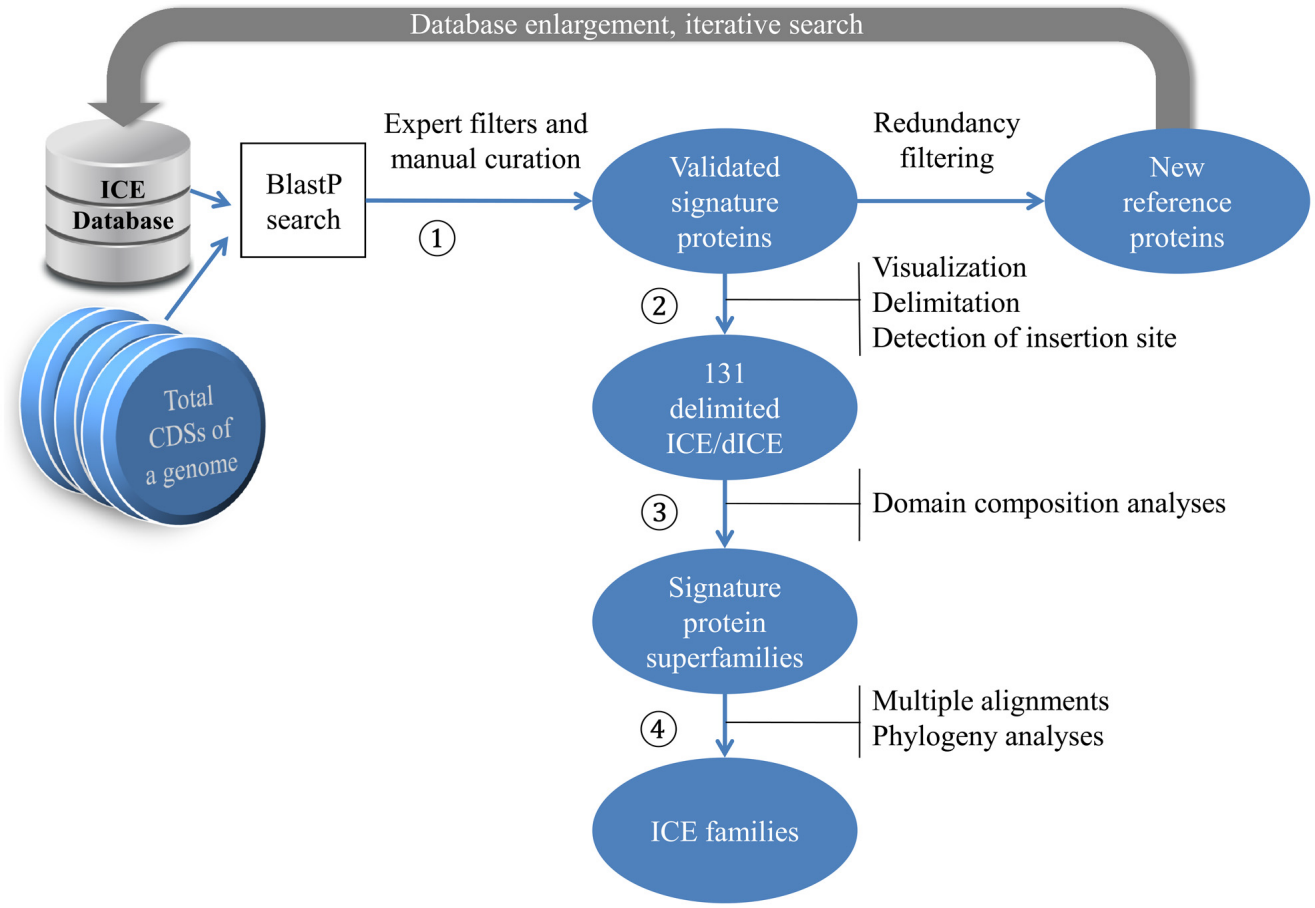
**Procedure for identifying candidate ICEs in sequenced genomes.** The amino-acid sequences encoded by a chromosome are collected as multifasta files and processed as follows (see Materials and Methods). AAA172 Signature proteins are identified by BlastP search using our reference sequences as query and the set of multifasta files as search database. Resulting hits are filtered and validated. AAA173 The location of genes encoding the validated signature proteins are visualized using Artemis and ICEs are delimited. AAA174 Domain composition of signature proteins are searched using Biomart and the signature proteins are grouped into classes. AAA175 Multiple alignments of signature proteins in each class are performed using Clustal Omega and their phylogeny is analyzed using maximum likelihood (ML) methods and BioNJ.

The overall workflow of our search strategy to detect and characterize ICEs in streptococcal chromosomes is depicted in **Figure 1**.

#### Detection of Signature Sequences in the Genomes of Streptococci

The first step of our workflow consists in the search for signature proteins (tyrosine integrase, serine recombinase or DDE transposase, CP, relaxase, and VirB4 proteins). It was performed by BlastP comparison, using the accelerated BlastP version implemented in the ngKlast software (Nguyen and Lavenier, 2009; with default parameters except for disabled low-complexity filter). The queries were the sequences of all reference ICE proteins and the target was the set of multifasta files (one per genome) representing all translated CDSs of the studied genomes. Expert filters were designed in the ngKlast system to remove hits corresponding to the translation of pseudogenes and to related proteins not involved in transfer or integration of conjugative elements (i.e., recombinases involved in inversion of DNA segments or in resolution of DNA molecule multimers, transcriptional regulators carrying a HTH DNA binding domain, DNA translocase FtsK involved in cell division, some toxins, etc). These filters include a percentage cover threshold (>25%), an E-value threshold (<1.10^-04^ for relaxases and integrases and <1.10^-05^ for CPs and VirB4) and a length threshold (>320 amino acids for integrases, >180 amino acids for relaxases, between 180 and 700 aa and between 1000 and 1200 aa for CPs and >500 amino acids for VirB4). Related proteins with biological function other than conjugation (i.e., XerS) passing through filters were manually removed. An iterative search was performed with the reference protein database enlarged with the newly found proteins and the same parameters until no new hit was found. Thus, only elements lacking significant sequence similarity with all signature proteins of ICE or carried by a plasmid could be missed. Sequence redundancy was eliminated using the BlastClust program with an identity threshold of 90%. The final reference database for ICE signature proteins contains non-redundant sequences from 106 tyrosine integrases, 43 serine integrases, 10 DDE transposases, 72 relaxases, 45 CPs, and 36 VirB4 proteins.

The relative positions of the CDSs corresponding to the detected signature proteins were visualized using Artemis (Rutherford et al., 2000). This step allowed checking if the CDSs co-localize in the genome and can thus be part of the same ICE. The detection of VirB4 guaranties the retrieval of ICEs rather than IMEs (integrative and mobilizable elements) that never encode this protein.

#### Detection of Insertion Sites and Delimitation of Putative ICEs

In the second step of the workflow (**Figure 1**), CDSs known to be potential insertion sites for ICE encoding site-specific integrases were searched and retained as potential candidates for insertion sites if located close to the CDSs of signature proteins. In their absence, insertion site was examined by comparing synteny with other genomes.

Putative ICEs were delimited by searching DRs on both sides of the putative ICEs by BLASTn analysis with either the 3′ end or the 5′ end of the potential insertion CDS or tRNA genes as a “query.” If no such potential insertion CDS was detected, the intergenic sequence downstream from the integrase gene was used as a query. ICEs closely related to Tn*916* were delimited by BLASTn with the ends of Tn*916* from *Enterococcus faecalis* DS16 as queries.

#### ICE/Decayed ICE (dICE) Counting

All the elements delimited with DRs and containing CDSs for the four complete signature proteins (integrase, relaxase, CP, and VirB4) were considered as ICEs. When some signature CDSs were missing or were incomplete, the complete CDS encoded by the closest ICE was compared to the putative defective one by tBlastN in order to detect possible genome annotation errors (e.g., mis-identification of an authentic gene as a pseudogene most frequently due to the presence of a type II intron within the gene or mis-identification of START codon suggesting truncated genes). Elements that carry one or two defective signature CDSs, or that lack one of its extremity were considered as decayed ICEs (dICEs).

#### Domain Composition Analysis

The third step of our workflow (**Figure 1**) involved retrieving domain composition of all ICE signature proteins from Uniprot annotations using the BioMart Central Portal^3^. *De novo* CD-search for conserved domains^4^ was performed when no data was available through BioMart.

#### Tree Construction

In this step (Step 4 on **Figure 1**), signature proteins were aligned using Clustal Omega with default parameters (Sievers et al., 2011). Trees of ICE signature proteins were built with MEGA (Tamura et al., 2013) using both maximum likelihood (ML; tree shown) based on JTT with Freqs (+F) model (partial deletion of gaps and missing data (80% cutoff), Gamma distributed with Invariant sites G+I (five categories)) and BioNJ methods with the Poisson model (Gouy et al., 2010). Branch support of the groupings was estimated using bootstrap (100 replicates for the ML method and 1,000 replicates for BioNJ).

#### ICE Annotation and Comparative Analysis

The comparative analysis of the conserved CDSs within a given ICE family was performed only for those that were non- or mis-characterized and displayed a significant number of ICEs. Functional annotation of ICEs was performed using Agmial (Bryson et al., 2006). Protein product, gene name and EC_number were assigned using similarity with Uniprot databank.

Data mining of the orthologs and the conserved syntenies was performed using Insyght. Sequence alignments were carried out at the protein level (using BLASTp) to achieve the pairwise comparisons of all the CDSs of ICEs belonging to the same family. Two genes were considered orthologous if they gave rise to a bi-directional best hit (BDBH) of the corresponding ICE genomic regions and if the sequence alignment included more than 50% of the total proteins with an e-value less than 0.01. Two CDSs for which the E-value of the sequence alignment was less than 0.01 were considered homologous but were not analyzed unless they belong to a synteny. Syntenies were computed with a dynamic programming algorithm that determines the highest scoring paths amongst the chains of colinear homologs. The scores and penalties used were as follows: minimum synteny score: 8; ortholog BBDH: 4; homolog non BDBH: 2; mismatch: -3; gap creation: -4; gap extension: -2. This setting allows the insertion of small gaps within the conserved synteny. The “Orthologs table” view in Insyght was used to identify the conserved and idiosyncratic loci within ICEs.

## Results

### Prevalence of ICEs and dICEs within Streptococcal Chromosomal Genomes

A total of 105 ICEs and 26 dICEs were identified among the 124 streptococcal genomes analyzed in this work (**Supplementary Table S1**). About half (63/124) of the examined strains contain at least one element and among those strains 61% (39/63) harbor several ICEs or dICEs. ICE denomination indicates whether the element is an ICE or a dICE, followed by letters and numbers allowing species and strain identification. When ICE/dICE encodes a site-specific integrase, its denomination also specifies the name of the target-gene. Otherwise, it indicates the integrase family (Tn*916* or DDE). For elements already well-characterized and named, the correspondence between names is indicated in **Supplementary Table S1**.

Some streptococcal species show relatively few ICEs and dICEs even if a significant number of genomes were analyzed (**Table 1**). In particular, in the *salivarius* group, only one element was found in the six analyzed genomes of *S. thermophilus* analyzed and no element in the three genomes of *S. salivarius*. By contrast, in the *anginosus* and *bovis* groups, there is an average of more than two ICEs or dICEs per genome, with extreme situations such as 13 and 11 elements found in the three genomes studied in *S. anginosus* and *S. gallolyticus*, respectively. The occurrence of ICEs and dICEs per species or strain can vary within a group. For example in the *pyogenic* group, there were only six elements found in the 19 *S. pyogenes* genomes analyzed but as many as 10 elements in the five *S. dysgalactiae* genomes analyzed.

**Table 1:** Prevalence of ICEs and dICEs within streptococcal species.

Group of species	*Streptococcus* species or strains	Number of strains	Total number of ICEs/dICEs per species	Minimum number of ICEs/dICEs per genome	Maximal number of ICEs/dICEs per genome	Average number of ICEs/dICEs per genome
anginosus	*S. anginosus*	3	13	2	7	4.3
anginosus	*S. intermedius*	3	7	1	3	2.3
anginosus	*S. constellatus*	3	6	0	4	2.0
bovis	*S. gallolyticus*	3	11	3	5	3.7
bovis	*S. pasteurianus*	1	3	3	3	NA
bovis	*S. infantarius*	1	2	2	2	NA
bovis	*S. lutetiensis*	1	2	2	2	NA
bovis	*S. macedonicus*	1	2	2	2	NA
mitis	*S. pneumoniae*	28	18	0	4	0.6
mitis	*S. oligofermentans*	1	2	2	2	NA
mitis	*S. mitis*	1	1	1	1	NA
mitis	*S. oralis*	1	1	1	1	NA
mitis	*S. pseudopneumoniae*	1	1	1	1	NA
mutans	*S. mutans*	4	1	1	1	0.3
pyogenic	*S. agalactiae*	8	13	0	7	1.6
pyogenic	*S. dysgalactiae*	5	10	0	5	2.0
pyogenic	*S. pyogenes*	19	6	0	3	0.3
pyogenic	*S. equi*	4	4	0	2	1.0
pyogenic	*S. parauberis*	1	2	2	2	NA
pyogenic	*S. iniae*	1	0	0	0	NA
pyogenic	*S. uberis*	1	0	0	0	NA
sanguinis	*S. parasanguinis*	2	3	1	2	1.5
sanguinis	*S. gordonii*	1	0	0	0	NA
sanguinis	*S. sanguinis*	1	0	0	0	NA
suis	*S. suis*	18	21	0	5	1.2
salivarius	*S. thermophilus*	6	1	0	1	0.2
salivarius	*S. salivarius*	3	0	0	0	0.0
ND	S. sp I-G 2	1	1	1	1	NA
ND	S. sp I-P16	1	0	0	0	NA


*NA, not applicable.*

### Diversity of ICE and dICE Relaxases, and VirB4 in Streptococci

A total of 121 relaxases, encoded by ICEs or dICEs, were detected. They can be classified in three distinct classes on the basis of their domains (**Table 2**). The ‘Rel-I’ regroups 52 relaxases that contain a C-terminal catalytic “Rep_trans” domain (PF02486) associated with an N-terminal Helix-Turn-Helix (PF01381) DNA binding domain. According to the CONJscan-T4SSscan server (^5^Guglielmini et al., 2011), these 52 relaxases belong to the MOB_T_ family that is related to initiators of rolling-circle replication of some plasmids and prophages (Guglielmini et al., 2014). The ‘Rel-II’ class regroups 62 relaxases sharing a common N terminal “relaxase” PF03432 catalytic domain and belonging to the MOB_P_ family. Among them, 20 relaxases also carry a C terminal “Lantibiotic streptin immunity” PF11083 domain of unknown function. The ‘Rel-III’ class of relaxases contains seven proteins with no identified domains according to CD-search. These proteins are classified by the CONJscan-T4SSscan server in the MOB_P_ family of relaxases.

**Table 2:** Characterization of the Conj_Tn*916*_, Conj_Tn*GBS2*_, and Conj_Tn*GBS1*_ superfamilies of conjugation modules.

Relaxases				Coupling proteins				VirB4				ICE/dICE superfamilies of conjugation modules		
			
Class	Pfam ID	Domain name	Conjscan	Class	Pfam ID	Domain name	Conjscan	Class^a^	Pfam ID	Domain Name	Conjscan	Name	ICEs	dICEs
Rel-I	PF02486	Rep trans	MOB_T_	CP-I	PF01580	FtsK_SpoIIIE	TcpA	VirB4-Ia	PF12846	AAA_10	VirB4	Conj_Tn*916*_	44	9
	PF01381	DNA binding						or Ib						
Rel-II	PF03432	Relaxase	MOB_P_	CP-IIa^b^	PF02534	T4SS-DNA_transfer	T4cp1/T4cp2	VirB4-Ic	PF12846	AAA_10	VirB4	Conj_Tn*5252*_	54	15
	PF11083 (optional)	Lantibiotic streptin immunity			PF12696	TraG-D_C								
Rel-III	None	None	MOB_P_	CP-IIb^c^	PF02534	T4SS-DNA_transfer	T4cp1/T4cp2	VirB4-Id	PF12846	AAA_10	VirB4	Conj_Tn*GBS1*_	7	2
					PF12696	TraG-D_C								


*^a^The four VirB4 subclasses are distinguished on the basis of their phylogenetic relatedness.*

*^b^598–688 aa.*

*^c^1045 aa.*

The 126 CPs encoded by ICEs or dICEs of streptococci are divided into two classes. The CP-I class groups 50 CPs sharing a unique central FtsK_SpoIIIE catalytic domain (PF01580; **Table 2**). According to the CONJscan-T4SSscan server, they belong to a particular class of CPs named TcpA, unrelated to all CPs of Gram-negative bacteria (Guglielmini et al., 2014). The ‘CP-II’ class contains 76 CPs with a central catalytic TraG/TraD domain (PF02534) and an additional C-terminal ‘TraM recognition site of TraD and TraG’ PF12696 domain. According to the CONJscan-T4SSscan server, these proteins belong to the main family of CPs named VirD4 (Guglielmini et al., 2014). Among them, 67 CPs, constituting the ‘CP-IIa’ class, are 598 aa to 688 aa-long. The nine remaining CPs are composed of about 1045 aa and are representatives of the ‘CP-IIb’ class (**Table 2**).

All 124 VirB4 proteins from ICEs and dICEs show a unique C-terminal PF12846 ‘AAA-10’ catalytic domain. Reconstruction of their phylogenetic relatedness (data not shown) suggested that they can be grouped in four classes designated ‘VirB4-Ia’ (30 proteins), ‘VirB4-Ib’ (20 proteins), ‘VirB4-Ic’ (65 proteins), and ‘VirB4-Id’ (nine proteins; **Table 2**). These proteins are all identified as VirB4 using the CONJscan-T4SSscan server.

### Classification of Conjugative Modules in Superfamilies and Families

**Table 2** summarizes the co-occurrence of the different classes of signature proteins within the conjugation modules of ICEs and dICEs. This allowed classification of the conjugation modules into three distinct superfamilies, named according to the first characterized element of each superfamily: Conj_Tn*916*_, Conj_Tn*5252*_, and Conj_Tn*GBS1*_.

#### The Conj_Tn*916*_ Superfamily of Conjugation Modules

The Conj_Tn*916*_ superfamily groups together 44 ICEs and nine dICEs. All encode a ‘Rel-I’ relaxase associated with a ‘CP-I’ and a ‘VirB4-Ia’ or ‘-Ib’ VirB4 protein.

**FIGURE 2:**
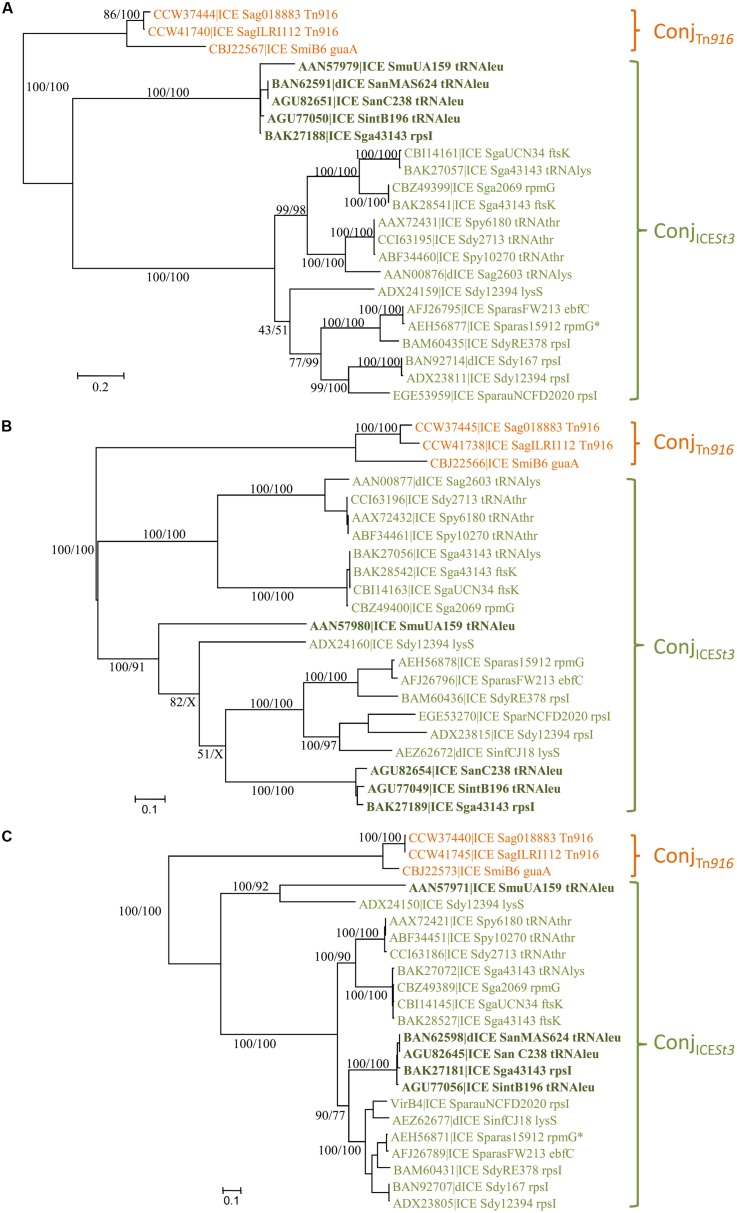
**Phylogenetic analysis of ICEs and dICE belonging to the Conj_Tn*916*_ superfamily.** **(A)** Relaxases; **(B)** coupling proteins; **(C)** VirB4 proteins. Bootstrap supports are given as followed: ML/BioNJ. X marks the nodes that are not validated with BioNJ. ICE names are colored according to the family of conjugation module they encode: orange = Conj_Tn*916*_, green = Conj_*ICESt3*_ (including elements encoding relaxases related to the one of ICE_*SmuA159_tRNAleu* in bold dark green). Because of their very close phylogenetic relationships, relaxases **(A)**, CP **(B)**, and VirB4 **(C)** of only three ICEs representative of the Conj_Tn*916*_ family are shown. Refer to **Supplementary Table S1** for ICE/dICE and strain details.

The phylogenetic tree of relaxases (**Figure 2A**) of the Conj_Tn*916*_ superfamily indicates three well-supported groups: relaxases related to the one of Tn*916*, to that of ICE_*SmuA159_tRNAleu* and to that of ICE*St3.* However, the latter two groups are not supported by the phylogenetic trees of CPs and VirB4 (**Figures 2B,C**). Therefore, two families of conjugative modules can be distinguished in the Conj_Tn*916*_ superfamily: the Conj_Tn*916*_ family and the Conj_ICE*St3*_ family.

The Conj_Tn*916*_ family is well-supported by phylogenetic trees of the relaxase, CP and VirB4 protein sequences (**Figures 2A–C**). It clusters 27 ICEs (and five dICEs) whose signature proteins are almost identical to those of the well-described Tn*916* element originally isolated from the Firmicute *E. faecalis* (Franke and Clewell, 1981). It also includes the more distant ICE_*SmiB6*_*guaA* whose signature proteins shared only 67, 66, and 80% identity with the *E. faecalis* Tn*916* relaxase, CP and VirB4 protein, respectively. All the 12 genes of the conjugation module of the prototype Tn*916* from *E. faecalis* DS16 were found with a similar organization in the vast majority of ICEs of the Conj_Tn*916*_ family (data not shown) thus confirming their relationships. Others only differ by one or few deletions, pseudogenizations or insertions.

The Conj_ICESt3_ family gathers together 21 elements whose signature proteins are much more variable in sequence than those of the Conj_Tn*916*_ family. The sequence of relaxases, CPs and VirB4 proteins of the most distantly related elements displayed around 20, 30, and 60% of identity, respectively.

**FIGURE 3:**
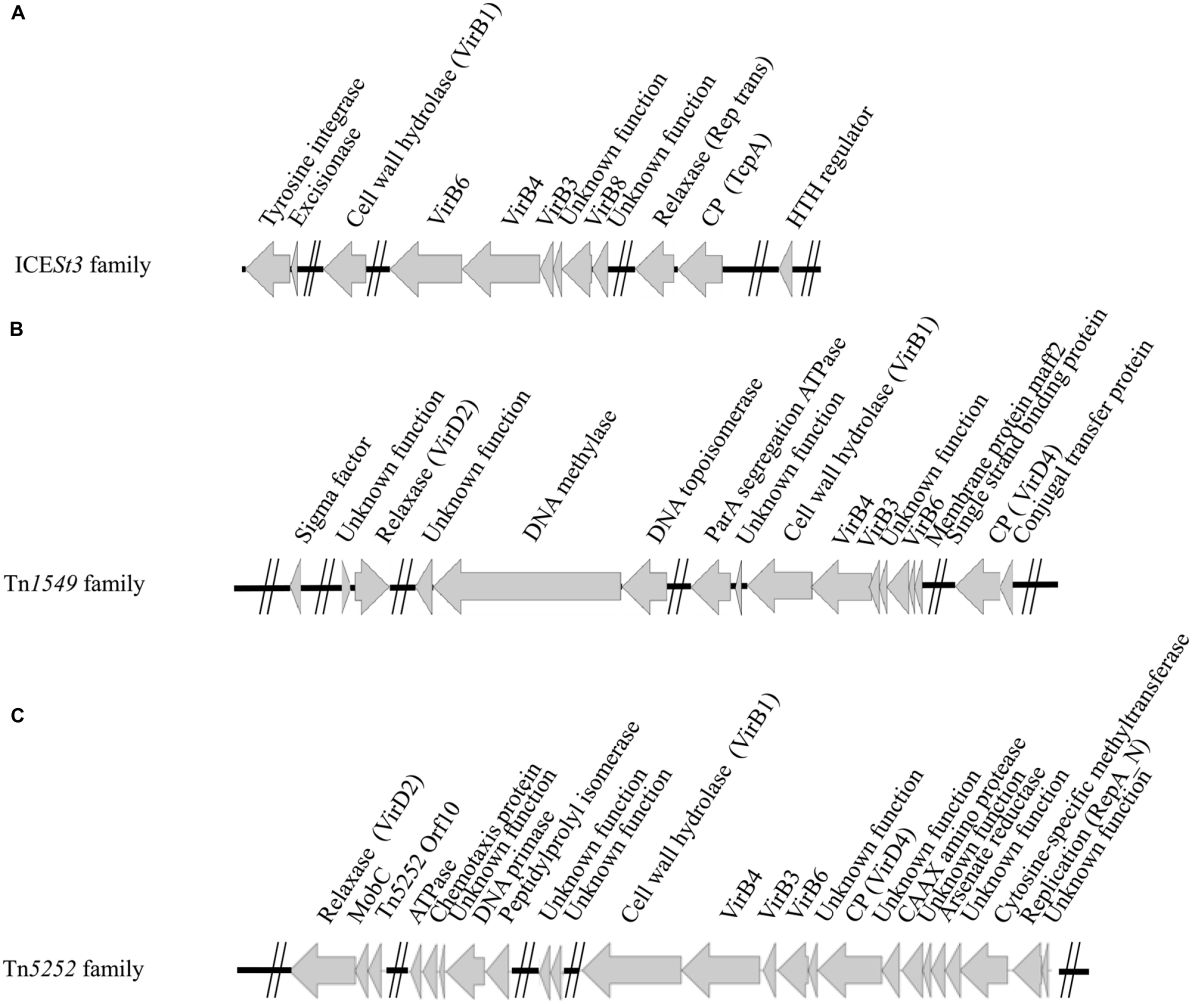
**Identification of conserved CDSs within the Conj_ICE*St3*_, Conj_Tn*1549*_ and Conj_Tn*5252*_ families of ICEs.** **(A)** Conj_ICE*St3*_ family; **(B)** Conj_Tn*1549*_ family; **(C)** Conj_Tn*5252*_ family. Genes encoding conserved proteins in all ICEs of a given family are indicated by arrows with below their putative function (or known homologs).

For a better characterization of the Conj_ICESt3_ family, a search for conserved CDSs in all members of this family was undertaken. Orthologous CDSs were identified on the basis of the identity of their product using Insyght. The integration module of all elements of the Conj_ICE*St3*_ family is composed of both a tyrosine integrase and an excisionase (**Figure 3A**). In addition to the relaxase, CP, VirB4 CDSs, tyrosine integrase and excisionase CDSs, they share seven other CDSs including CDSs involved in the T4SS formation (VirB1, VirB3, VirB6, and VirB8) and a CDS encoding a regulation protein (HTH regulator).

#### The Conj_Tn*5252*_ Superfamily of Conjugation Modules

**FIGURE 4:**
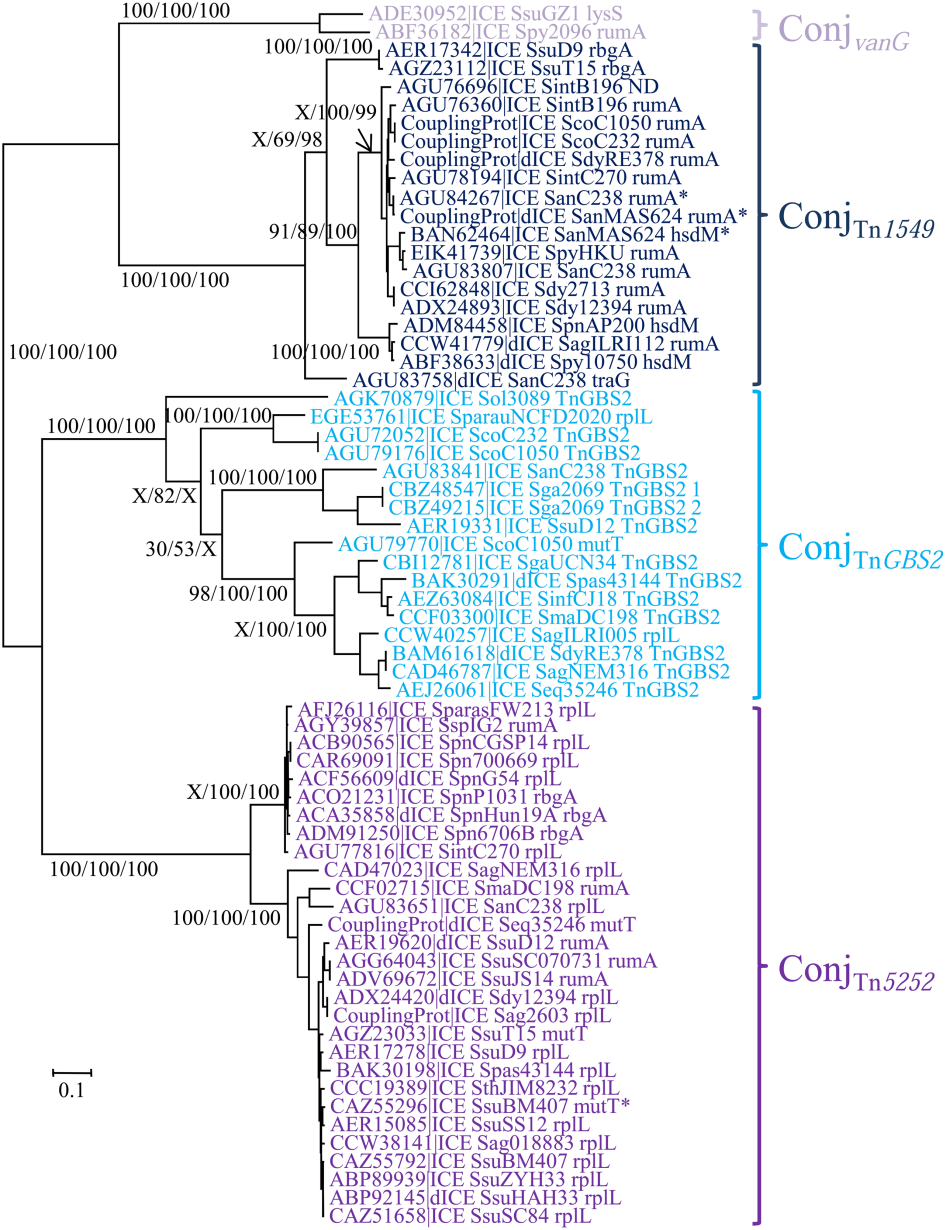
**Phylogenetic analysis of ICEs and dICEs belonging to the Conj_Tn*5252*_ superfamily.** The trees of CPs are shown in this figure. ML boostrap values for relaxases/CPs/VirB4 (in this order) are given. X marks the nodes that are not validated with other proteins. Families are also supported by BioNJ analysis (data not shown). In mauve are ICEs/dICEs belonging to the Conj_*vanG*_ family, in dark blue those belonging to the Conj_Tn*1549*_ family, in light-blue those of the Conj_TnGBS2_ family and in purple that of the Conj_Tn*5252*_ family. Elements marked with an asterisk are not integrated in their primary sites but in secondary ones. Refer to **Table 1** for ICE/dICE and strain details.

The Conj_Tn*5252*_ superfamily gathers together 54 ICEs and 15 dICEs encoding a ‘Rel-II’ relaxase associated with a ‘CP-IIa’ CP and a ‘VirB4-IIc’ VirB4 (**Table 2**). The phylogenetic trees obtained independently for ‘Rel-II’ relaxases, ‘CP-IIa’ CPs and ‘VirB4-Ic’ proteins (**Figure 4**) are congruent and therefore only the CP one is shown. These data are consistent with the splitting of the Conj_Tn*5252*_ superfamily into four distinct families: Conj_v*anG*_, Conj_Tn*1549*_, Conj_Tn*GBS2*_, Conj_Tn*5252*_ that cluster 2, 21, 17, and 29 elements, respectively.

As expected from Guérillot et al. (2013), sequence comparison of the 15 ICEs encoding a Conj_Tn*GBS2*_ module identified 14 CDSs shared by all these Tn*GBS2*-related elements (data not shown).

Using Insyght, CDSs comparison of the 14 ICEs of the Conj_Tn*1549*_ family identified 14 CDSs shared by all of them, in addition to the relaxase, CP and VirB4 CDSs (**Figure 3B**). One of these CDSs encodes a sigma factor that may be involved in the regulation of ICE transfer; three others being probably involved in the T4SS formation (VirB1, VirB3, VirB6) and one in maintenance of the ICE after excision (segregation ATPase). Many of these ICEs also encode a protein carrying a repA_N domain that could be involved in maintenance of excised ICEs.

As for ICEs of the Conj_Tn1*549*_, the 23 ICEs of the Conj_Tn*5252*_ family do not share the same integration module. However, they display 24 conserved CDSs. In addition to the relaxase, and the CP VirB4 CDSs, they share three other CDSs probably involved in the T4SS formation (VirB1, VirB3, VirB6), and one encoding a replication initiator (repA_N; **Figure 3C**).

#### The Conj_Tn*GBS1*_ Superfamily of Conjugation Module

The Conj_Tn*GBS1*_ superfamily of conjugation modules is the least represented in streptococcal genomes with only seven ICEs and two dICEs exhibiting such a module. It is characterized by the presence of a ‘Rel-III’ relaxase co-occurring with a ‘CP-IIb’ CP and a ‘VirB4-Id’ protein. This superfamily was not identified by Guglielmini et al. (2014).

**FIGURE 5:**
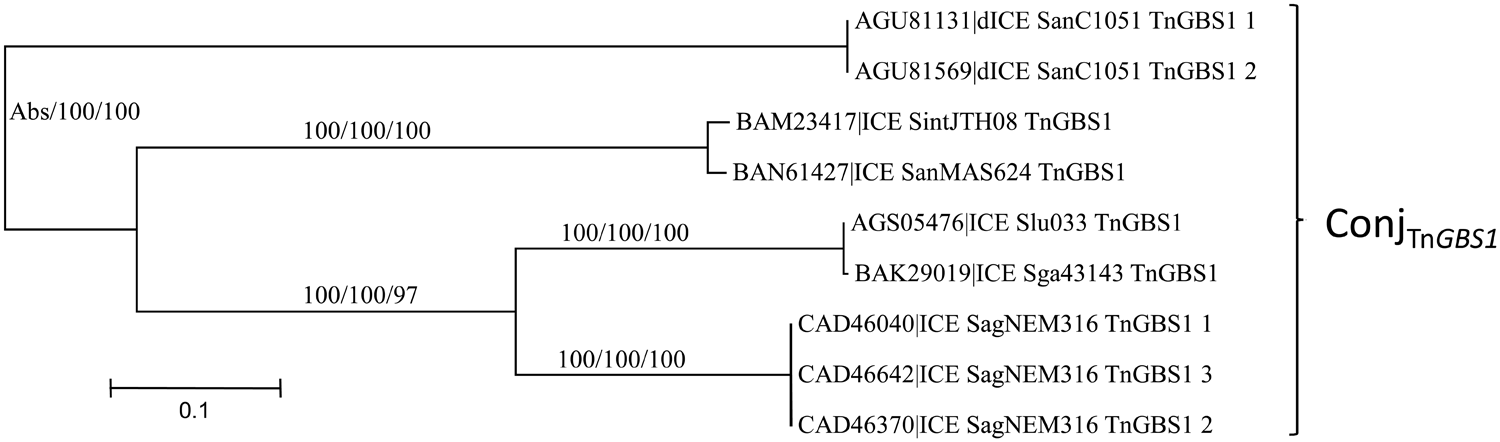
**Phylogenetic analysis of ICEs and dICEs belonging to the Conj_Tn*GBS1*_ superfamily.** The trees of CPs are shown in this figure. ML boostrap values for relaxases/CPs/VirB4 is given in this order. Families are also supported by BioNJ analysis (data not shown). Abs means that one sequence was missing. Refer to **Supplementary Table S1** for ICE/dICE and strain details.

As for the Conj_Tn*5252*_ superfamily, whatever the signature protein used, all phylogenetic trees are congruent and therefore only the CP one is shown (**Figure 5**). Sequence comparison of the signature proteins of the most divergent ICEs of this superfamily indicated that they shared more than 56% of identity. Thus, within streptococcal genomes, the Conj_Tn*GBS1*_ superfamily is represented by the unique Conj_Tn*GBS1*_ family. As expected from Guérillot et al. (2013), systematic comparisons of the protein sequence of the members of the Conj_Tn*GBS1*_ family with Tn*GBS1* CDSs confirmed that they are related to Tn*GBS1* (data not shown).

### Prevalence of the Different Families of Conjugation Modules within Streptococcal Species

In summary, seven families of conjugation modules belonging to three superfamilies were identified in streptococcal ICEs/dICEs. Most ICEs of the Conj_Tn*916*_ family are found in *S. suis* and *S. pneumoniae*, while *S. dysgalactiae* and *S. gallolyticus* mainly harbor ICEs of the Conj_ICE*St3*_ family. ICEs of the ConjTn_*5252*_ family are frequently found in *S. suis*, and ICEs of the Conj_TnGBS1_ family are only found in *S. agalactiae*, *S. anginosus*, *S. intermedius*, and *S. lutetiensis* genomes.

### Diversity of Integration Modules and Integration Sites of Streptococcal ICEs and dICEs

Three unrelated families of integrases (tyrosine integrase, serine integrase, and DDE transposase) are encoded by streptococcal ICEs/dICEs. Most of these integrase genes are located at one extremity of the ICE and adjacent to the integration site.

#### Prevalence and Integration Site of ICEs/dICEs Encoding a Tyr Integrase

Tyrosine integrases are the most prevalent integrases detected as they are found in 53% of the elements. In total, 73 tyrosine integrases were identified (66 for ICEs and 7 for dICEs). Most of them are ∼400 aa long, except for five that are composed of 502 aa. Despite a variable degree of identity between these proteins, all tyrosine recombinases share a “phage-integrase” PF00589 domain in their N-terminal region. Most of them carry an additional N-terminal binding domain being either: (i) a pfam02920 “DNA binding domain characteristic of Tn*916* integrase” or (ii) a PF14659 “Phage integrase, N-terminal SAM-like domain” (iii) and/or more rarely a PF14657 “AP2-like DNA-binding integrase domain.”

**FIGURE 6:**
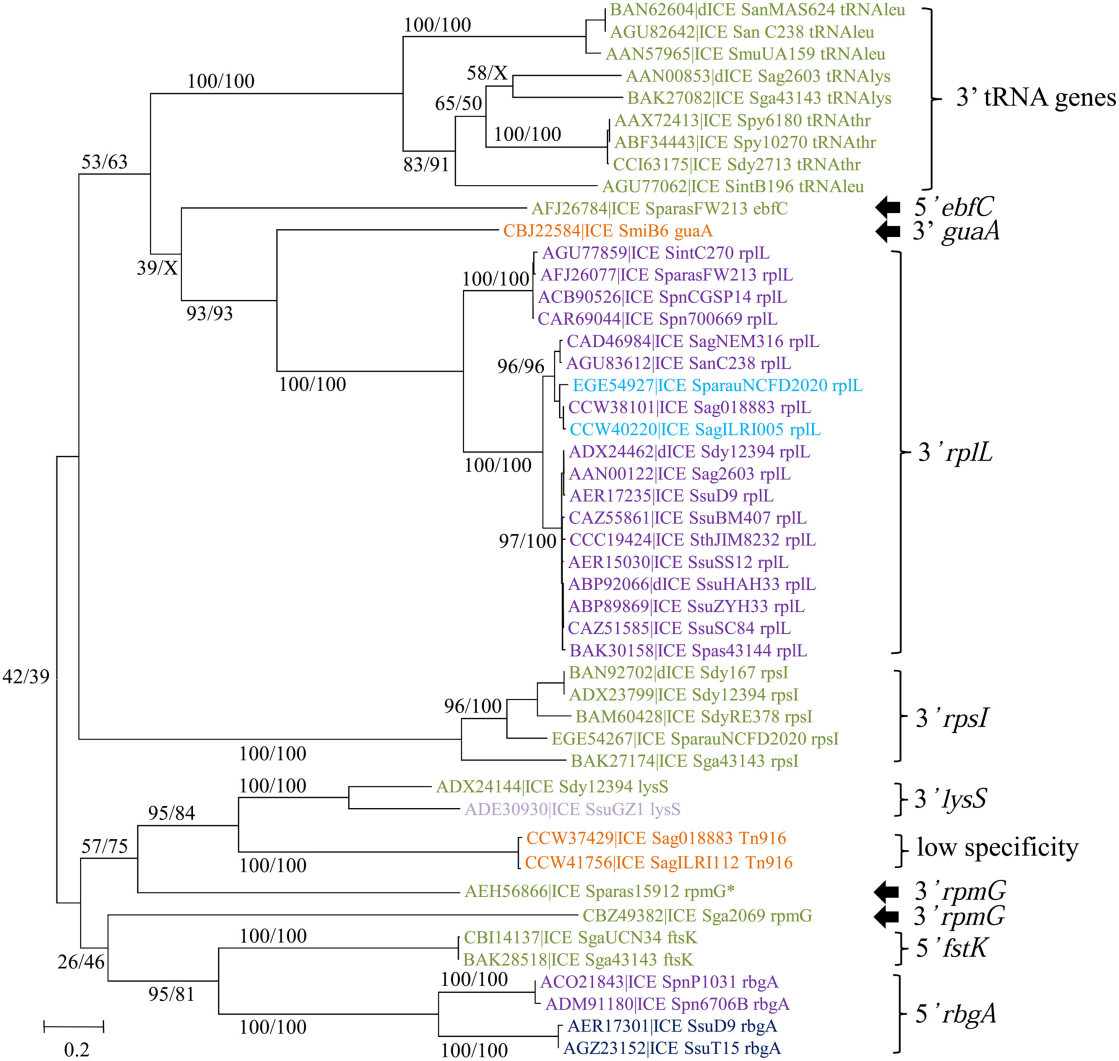
**Phylogenetic trees of tyrosine integrases from streptococcal ICEs/dICEs.** Bootstrap values are given as followed: ML/BioNJ. X marks the nodes that are not validated with BioNJ. ICE names are colored according to the family of conjugation module they encode: orange = Conj_Tn*916*_, green = Conj_ICE*St3*_, mauve = Conj_*vanG*_, dark-blue = Conj_Tn*1549*,_ purple = Conj_Tn*5252*_, and in light-blue those of the Conj_Tn*GBS2*_. Brackets gather together closely related integrases sharing the same specificity of integration. Integration specificity of the ICEs/dICEs is indicated. Elements marked with an asterisk are not integrated in their primary sites but in secondary ones. Refer to **Supplementary Table S1** for ICE/dICE and strain details.

The phylogenetic tree of the tyrosine integrases reveals 11 well-supported groups (**Figure 6**). The overall finding is that almost all tyrosine integrases are grouped according to their insertion loci. Exceptions are the two non-grouped tyrosine integrases catalyzing integration in the 3′ end of the *rpmG* gene that display two distinct orientations and locations relative to the *rpmG* gene (**Figure 6**) and share only 21% of identity. Interestingly, all tyrosine recombinases catalyzing integration in a tRNA gene are grouped together. Half of the elements encoding a tyrosine integrase are inserted in the 3′ end of either tRNA CDSs (9/73) or a well-conserved housekeeping genes (30/73) such as *rplL* (L7/L12 ribosomal protein)*, rpsI* (S9 ribosomal protein)*, lysS* (lysyl-tRNA synthetase)*, rpmG* (L33 ribosomal protein), or *guaA* (GMP synthase). In rare cases (7/73), the integration sites are found in the 5′ end of genes such as *ftsK* (DNA translocase involved in cell division), *rbgA* (ribosomal biogenesis GTPase) and *ebfC* (nucleoid associated protein). The remaining tyrosine integrases (27/73) catalyze low-specificity integration as previously shown for the very closely related integrase of Tn*916* (Scott et al., 1994).

**FIGURE 7:**
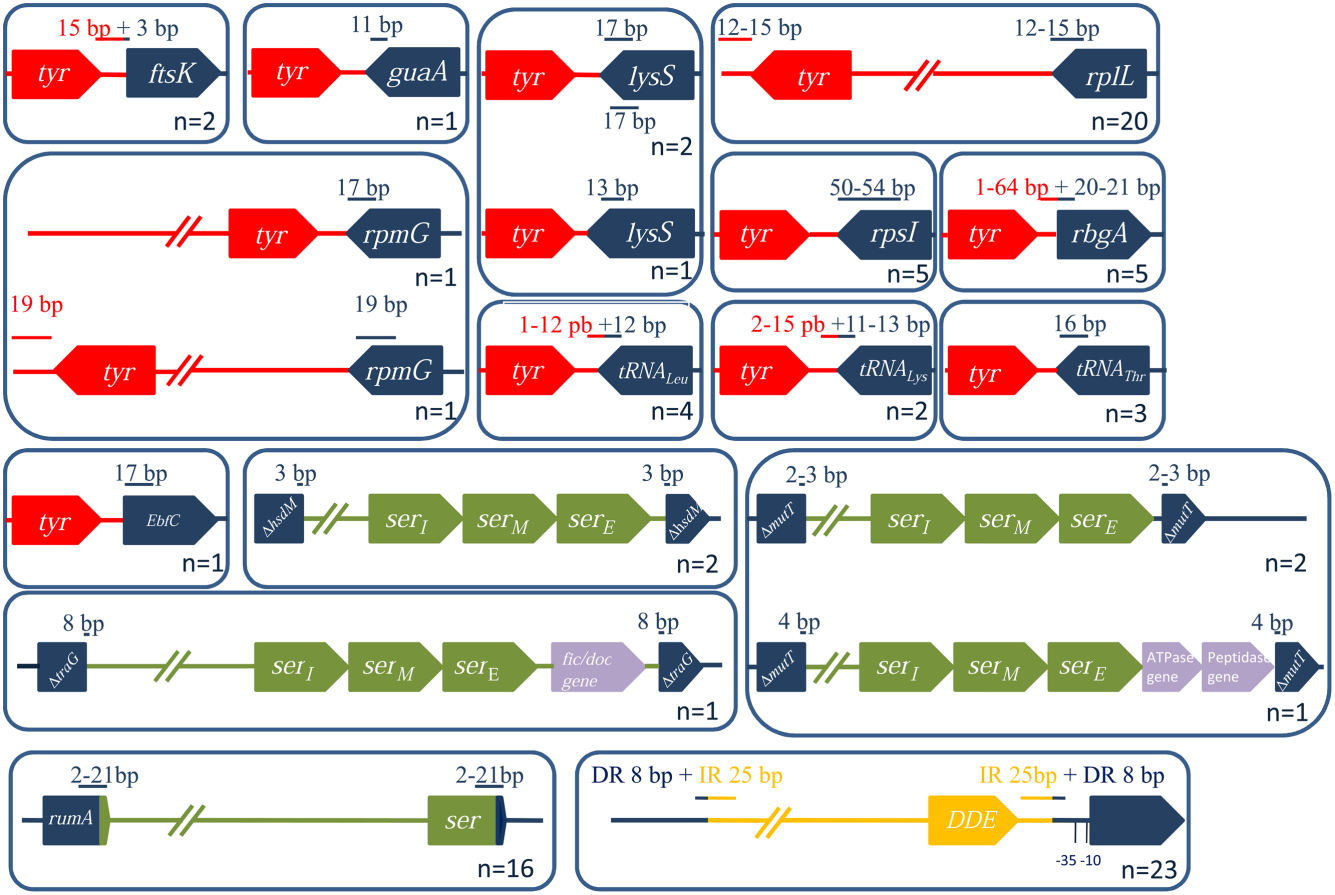
**Characterization of ICE/dICE integration loci and their position relative to the integrase CDSs.** The genes within (or next to) which an ICE is inserted are in blue. Tyrosine integrases are in red, serine recombinases in green and DDE transposases in yellow. The sizes (in bp) of the DR (or of IRs when specified) are indicated in red when the sequence is inside of the conjugative element (in blue, outside). Numbers represent the number of ICEs integrated in a given target gene.

All the ICEs/dICEs encoding a site-specific tyrosine integrase are flanked by DRs whose size ranges from 12 to 54 bp. DRs in *rplL, guaA* or in genes encoding tRNAthr only contain the exact 3′ end of the target genes. By contrast, DRs of elements integrated in the 5′ end of *ftsK* and *rbgA*, and in the 3′ end of genes of tRNAleu and tRNAlys, overlap the flanking intergenic regions (**Figure 7**). Three elements are integrated in the *lysS* gene and are flanked by DRs with similar sequences. However, these DR sequences have distinct locations within *lysS* resulting from a slight difference in the length of this gene: one corresponds to the last 17 bp of the *lysS* gene while the others contain 13 bp and are more internal (**Figure 7**).

#### Prevalence and Integration Sites of ICEs and dICEs Encoding a Serine Integrase

A total of 42 serine integrases were identified: 32 from ICEs and 10 from dICEs. They all displayed an N-terminal catalytic and dimerization ‘Resolvase’ domain (PF00239) that contains a conserved serine residue. This domain is always associated with a ‘Recombinase’ PF07508 domain. All of them but 10 also contain a pfam13408 ‘Zinc ribbon recombinase’ domain that is likely to play a DNA-binding role.

**FIGURE 8:**
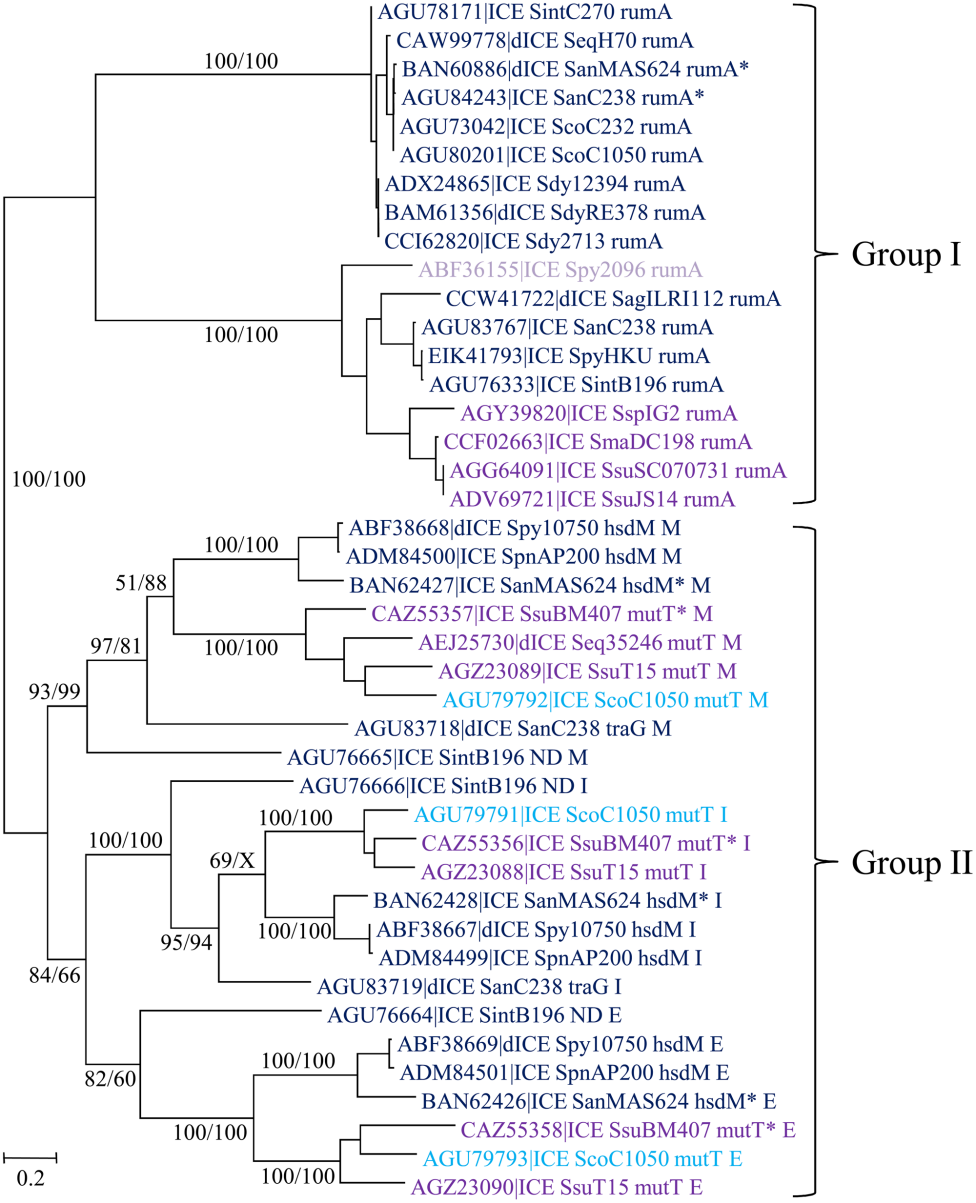
**Phylogenetic tree of serine integrases from streptococcal ICE/dICE.** Bootstrap values are given as followed: ML/BioNJ. X marks the nodes that are not validated with MP. ICE name are colored according to the family of conjugation module they encode (dark-blue = Conj_Tn*1549*_, purple = Conj_Tn*5252*_, mauve = Conj_*vanG*_), and in light-blue those of the Conj_Tn*GBS2*_. Brackets gather together closely related integrases sharing the same specificity of integration. Genes in which the ICEs/dICEs are integrated are indicated. Refer to **Supplementary Table S1** for ICE/dICE and strain details. Elements marked with an asterisk are not integrated in their primary sites but in secondary ones.

Streptococcal conjugative elements encode either a single serine integrase (14 ICEs and four dICEs) or a triplet of serine integrase genes (6 ICEs and 3 dICEs). The phylogenetic tree of the serine integrases is compatible with the existence of two groups of integrases (**Figure 8**). One of the groups clustered all the single serine integrases that target integration in *rumA* [23S rRNA (uracil-5-)methyltransferase]. ICEs/dICEs encoding such serine integrase are flanked by 2–21 bp-DRs localized at one end in the *rumA* gene and at the other end in the serine integrase gene (**Figure 7**). Integration leads to a reciprocal exchange of the 3′ part of the *rumA* and serine integrase genes leading to a modification of sequence and length of the C-terminal end of the corresponding proteins. It should be noticed that the replacement changes the translation frame of both proteins.

The second group of serine integrases is composed of the 24 integrases that are organized in triplets within the elements (**Figure 8**). Interestingly, these serine integrases are clustered according to their position within the triplets: external (E), middle (M), or internal (I) with respect to the ICE/dICE extremity (**Figure 8**). This suggests that all these modules derive from an ancestral module that already encoded three serine recombinases and that the presence of triplet results from two successive duplications. Triplets of serine integrases catalyze site-specific integration within several genes thus leading to their disruption: *mutT* (Nudix hydrolase), *traG* (CP of another ICE, dICE, or ICE remnant) and *hsdM* (methyltransferase subunit of type I restriction-modification systems). Insertion of ICEs/dICEs encoding triplets of serine integrases leads to very small 2–8 bp DRs (**Figure 7**). In some cases, one or several CDSs separated the triplets of serine integrases from the target gene (**Figure 7**). All serine integrases have the same orientation with respect to the target gene.

In several cases (element names marked with an asterisk in **Figure 8**), the comparison of the observed integration sites with the ones of elements with closely related integrases strongly suggests that these elements are not integrated in their primary sites but in secondary ones. ICE_*SanMAS624_hsdM*^∗^ disrupts a gene encoding a protein carrying the domain PF0267 (“Adenine nucleotide alpha hydrolase”). ICE_*SsuBM407_mutT*^∗^ disrupts a gene encoding a luciferase-like protein. dICE_*SanMAS624_rumA*^∗^ and ICE_*SanC238_rumA*^∗^ disrupts *sstT*, a gene encoding a serine/threonine transporter. The relaxase, CP and VirB4 of these last two elements are very closely related (**Figure 4**) as well as their integrases (**Figure 8**). This suggests that they were both inherited from the last common ancestor of their hosts.

#### Prevalence and Integration Sites of ICEs/dICEs Encoding a DDE Transposase

**FIGURE 9:**
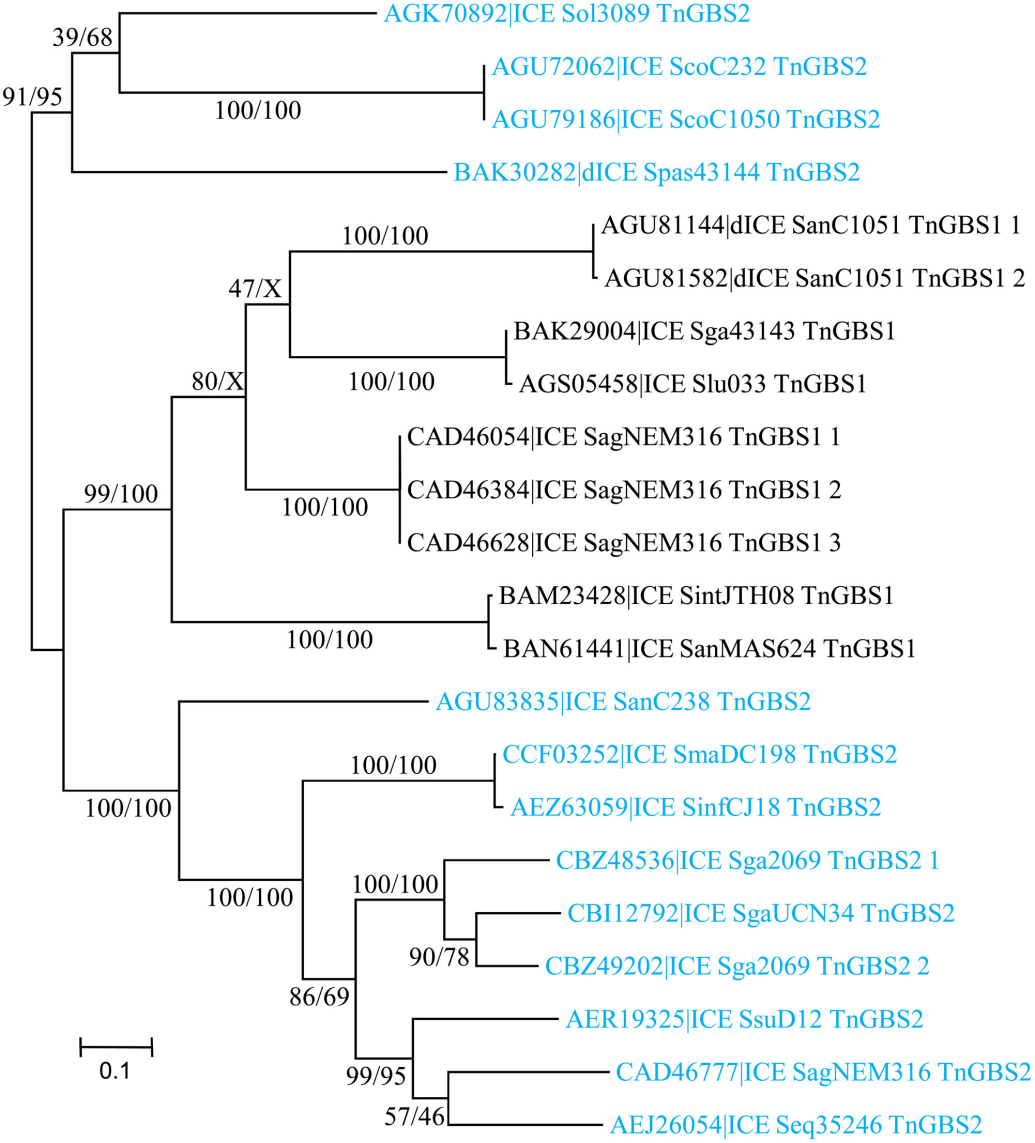
**Phylogenetic tree of DDE transposases from streptococcal ICE/dICE.** Bootstrap values are given as followed: ML/BioNJ. ICE name are colored according to the family of the conjugation module they encode (black = Conj_Tn*GBS1*_, light-blue = Conj_Tn*GBS2*_. Refer to **Table 1** for ICE/dICE and strain details.

A total of 23 DDE transposases related to those encoded by Tn*GBS1* and Tn*GBS2* elements (Guérillot et al., 2013) were detected. All show an “Uncharacterized protein family” PF06782 conserved domain with unknown function. Alignment of the amino acid sequences of the most divergent DDE transposases revealed that they share 42% identity showing that all these transposases belong to the same family. However, DDE transposases encoded by ICEs carrying a Conj_Tn*GBS1*_ module are clustered together and are distinct from those encoded by ICEs with a CONJ_Tn*GBS2*_ module (**Figure 9**).

Analysis of the junction sequence of ICEs encoding DDE transposases shows an 8-bp DR sequence that results from the duplication of the target sequence. Comparison of the insertion sites between all ICEs and dICEs of this group did not reveal any significant sequence similarity among the duplicated sequences but they are all located 15–16 pb upstream from -35 boxes of sigma A promoters as previously reported (Brochet et al., 2009).

### Diversity and Evolution of ICEs/dICEs

The determination of the limits of each element allows the comparison of their size. If one excludes the Conj_*vanG*_ ICE family (only two elements), the Conj_Tn*916*_ and Conj_Tn*GBS1*_ families are the most homogeneous in size with elements from 18 to 26 kb and from 40 to 53 kb, respectively. By contrast, the size of the Conj_ICE*St3*_ elements is much more variable: most of them are 19- to 37-kb long and one exceeds 60 kb. The size of the Conj_Tn*1549*_ elements can double (from 36 to 72 kb) and that of Conj_Tn*GBS2*_ elements shows a very large disparity (from 25 to 82 kb). When ICEs/dICEs are present within *Streptococcus* genomes, they contribute to 1–13% of chromosomal DNA.

**FIGURE 10:**
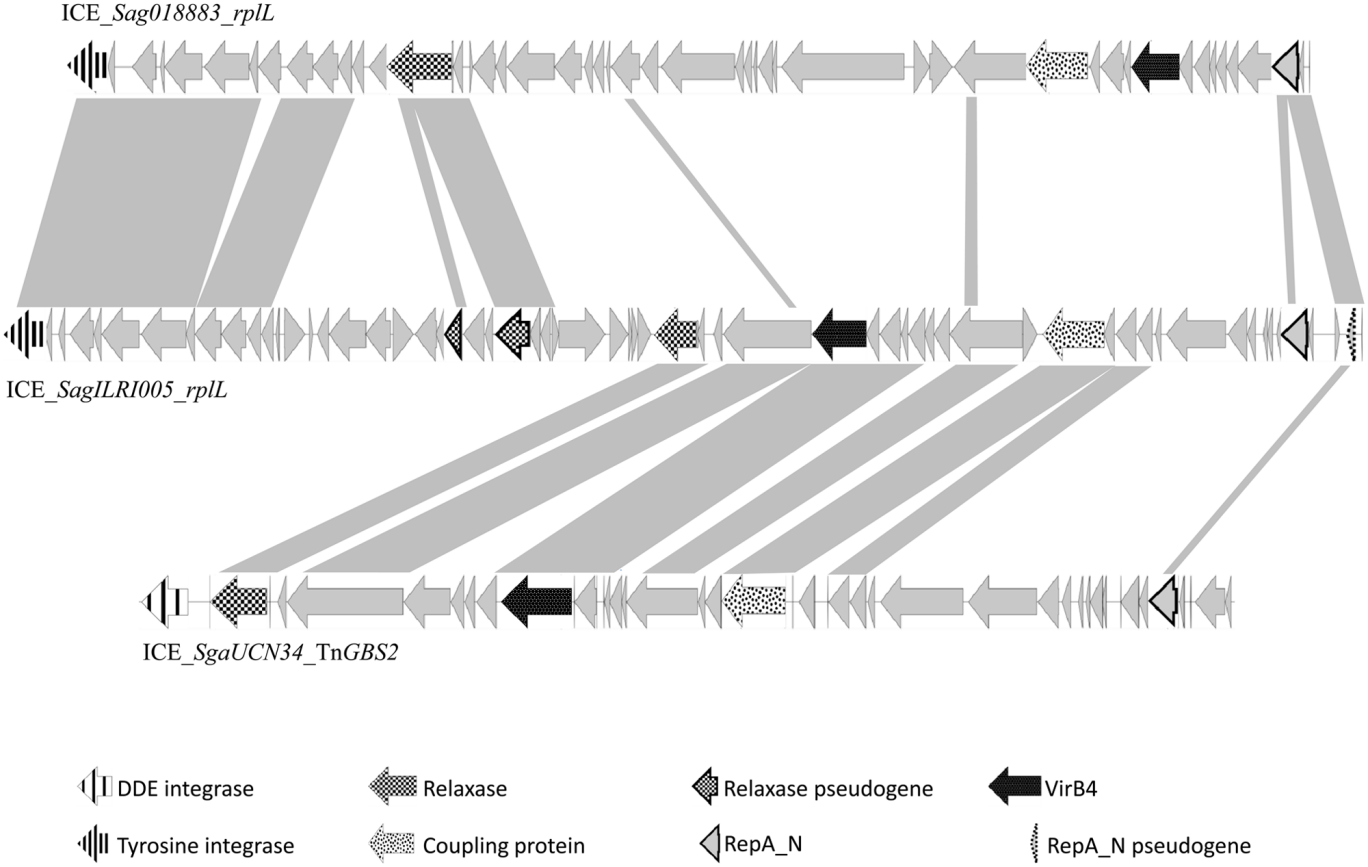
**Genetic organization of ICE_*SagILRI005_rplL* and comparison against ICE_*SgaUCN34_TnGBS2* and ICE_ *Sag018883_rplL*.** For the comparison, gray shading between the genetic elements represents regions with >50% amino acid sequence identity. The arrows represent the individual ORFs. Putative functions of the conserved genes are those deduced from the functional annotation of the ICE using Agmial.

Analysis of streptococcal ICEs and dICES also allowed determining their typical combination of conjugation and integration/excision modules (**Table 3**). Almost all conjugation modules of the Conj_Tn*916*_ family are associated with a tyrosine integrase identical, or almost identical, to the one of Tn*916*, that is known to have a low specificity of integration (Scott et al., 1994). The only exception is ICE_*SmiB6_guaA* that encodes a conjugation module of the Conj_Tn*916*_ family but is site-specifically integrated in the 3′ end of *guaA*. Elements with a Conj_ICE*St3*_ module are associated with site-specific tyrosine integrases catalyzing integration in eight distinct integration sites (3′ end of three types of tRNA encoding genes, *rpsI*, *rpmG*, and *lysS* as well as the 5′ end of *ftsK* or *ebfC*). The conjugation modules of Conj_Tn*5252*_ superfamily can be associated with a tyrosine recombinase, a serine site specific recombinase or a DDE transposase. Thus, ICEs and dICEs carrying a Conj_Tn*1549*_ module are associated with: (i) a single serine integrase catalyzing the insertion in *rumA*; (ii) a triplet of serine integrases catalyzing the insertion in *hsdM* or *traG* or (iii) a tyrosine integrase catalyzing the insertion in the 5′ end of *rbgA*. Those carrying a Conj_Tn*5252*_ module can encode: (i) a tyrosine integrase catalyzing the integration in the 3′ end of *rplL* or the 5′ end of *rbgA*; (ii) a single serine integrase catalyzing the integration in *rumA*; (iii) or a triplet of serine integrases catalyzing the insertion in *mutT*. The two ICE (ICE_*SsuGZ1_lysS* and *ICE_Spy2096_rumA*) displaying a Conj_vanG_ conjugation module are associated with a tyrosine or a serine integrase, respectively. All the Conj_Tn*GBS1*_ and 14 of the 17 Conj_Tn*GBS2*_ conjugation modules are associated with a DDE transposase. However, three ICEs carrying a Conj_TnGBS2_ module are not associated with a DDE transposase: ICE_*Sco1050_mutT* encodes a triplet of serine recombinases and both ICE_*SparauNCFD2020_rplL* and ICE_*SagILRI005_rplL* encode a tyrosine integrase. Sequence comparison suggests that ICE_*SagILRI005_rplL* is composite (**Figure 10**). Its Conj_TnGBS2_ conjugation module is closely related to the one of ICE_*SgaUCN34_TnGBS2*. However, its left part is closely related to the left part of ICE_*Sag018883_rplL*, an ICE carrying a conjugation module belonging to CONJ_Tn*5252*_ family. It includes not only the recombination module but also a lactose utilization module and a pseudogene of relaxase typical of the Conj_Tn*5252*_ family. The right end of ICE_*SagILRI005_rplL* carries a gene encoding a repA_N domain closely related to a gene located in the right of ICE_*Sga018883_rplL* and additionally a pseudogen, whose product also carry a repA_N domain.

**Table 3:** Various combinations of conjugation and integration modules in ICEs and dICEs.

Conjugation module		Number of elements	Integrases or transposases			
		
Superfamily	Family		Tyrosine	Serine (Single)	Serine (Triplet)	DDE
Conj_Tn*916*_	Conj_Tn*916*_^1^	32	28	0	0	0
	Conj_ICE*St3*_	21	21	0	0	0
Conj_Tn*5252*_	Conj_Tn*5252*_^*2*^	29	19	4	3	0
	Conj_Tn*1549*_^*3*^	21	2	13	5	0
	Conj_TnGBS2_	17	2	0	1	14
	Conj_*vanG*_	2	1	1	0	0
Conj_Tn*GBS1*_	Conj_TnGBS1_	9	0	0	0	9


*^1^Four Conj_Tn*916*_ elements are deprived of an integrase CDS (absent or as a pseudogene).*

*^2^Three Conj_Tn*5252*_ elements are deprived of an integrase CDS (absent or as a pseudogene).*

*^3^One Conj_Tn*1549*_ element exhibits an integrase pseudogene.*

## Discussion

### Detection of ICEs and dICEs in Streptococcal Genomes

Burrus et al. (2002a), a precursor analysis of 24 genomes from various Firmicutes, led to the identification of 17 putative ICEs and suggested that these elements are widespread at least in this division of bacteria. Over the last decade, with the revolution of sequencing technology, the number of fully sequenced bacterial genomes greatly increased. By the same time, efforts were made to improve *in silico* analysis of the data sets and in particular those allowing the detection of genomic islands including ICEs. Almost all searches of ICEs in bacterial genomes were only performed with a strain-centric or an ICE family centric point of view. However, few recent studies also reported extensive characterization of these conjugative elements. The first extensive study identified 335 chomosomal conjugative modules by scanning 1124 genomes of prokaryotes for conjugative genes (using HMM profiles of conjugative proteins of essentially proteobacterial plasmids; Guglielmini et al., 2011). Soon after, Ghinet et al. (2011) reported the characterization of 161 ICEs within 275 genomes of Actinobacteria but did not identify their limits. More recently, Puymège et al. (2015) searched for genetic elements integrated in the tRNAlys _CTT_ gene in 303 genomes of *S. agalactiae*, leading to the identification and delimitation of 108 putative ICEs or derivatives. It should be noticed that in 2012, Bi et al. (2012) developed a web database^6^, compiling information on ICEs from both Gram^+^ and Gram^-^ bacteria. However, if this database has the merit to list a large number and a great diversity of elements, it was not updated since November 2012 and some studies describing novel streptococcal ICEs published before this date escaped to the attention of the authors (for example, Brochet et al., 2008, reporting 10 novel ICEs). ICEberg have limitations considering ICEs from *Streptococcus* since (i) about half of ICE/dICE boundaries are incorrectly delimitated in ICEberg and (ii) the insertion site of many of them, although published, is not registered in this database. More widely, information on numerous elements from Firmicutes is inconsistent or wrong. Thus, while the authors indicated that a family should include only elements that carry both related integration and conjugation modules, they also included in the Tn*916* family ICEs that carry Tn*916*-unrelated integration modules (such as Tn*5397* or ICE*Lm1*), Tn*916*-unrelated conjugation modules (such as Tn*1549*) or elements completely unrelated to Tn*916* but carrying conjugation modules related to the one of Tn*1549* (such as CTn*2* or CTn*5*). Furthermore, although ICE*Lm1* and Tn*5801* carry almost identical integration and conjugation modules (Burrus, plasmid 2002, cited in ICEberg for ICE*Lm1*), they were included in different families, Tn*916* and Tn*5801*, respectively. Even more problematically, the Tn*1207.3* family and 10750-RD.1 family only contain elements unrelated to ICEs, prophages for the first one and highly decayed derivates of integrative mobilizable elements for the second one. In general, these failures (and many others not mentioned here) make this database unreliable and very difficult to use for ICEs from *Streptococci* and other Firmicutes.

Here, we present the results of ICE detection within 124 complete streptococcal genomes. Our search is based on an iterative search for genes encoding signature proteins from ICEs. The co-occurrence of an integrase and three proteins of the conjugation module guaranties the retrieval of ICEs or dICEs. When one or two signature CDSs appeared to be a pseudogene or to be absent, an analysis of the whole element was undertaken to confirm its nature.

This work led to the identification and characterization in 63 *Streptococcus* genomes of 131 ICEs/dICEs whose extremities were precisely mapped on the genome. Elements that were already precisely identified are marked by a reference in **Supplementary Table S1**.

### Distribution of ICEs and dICEs in the Different Streptococcal Species

Among the 27 streptococcal species analyzed, all, except 5 (*S. iniae*, *S. gordonii*, *S. uberis, S. sanguinis*, for which only one complete genome is available and *S. salivarius* for which three genome sequences exist), contain ICEs/dICEs showing the ubiquity of these elements in *Streptococcus*. *S. suis* appears as the species containing the highest number of ICEs/dICEs since 61% of the strains carry at least one ICE/dICE. However, the high prevalence of ICEs/dICEs in this species might be due to strain sampling since many strains carrying an ICE are related. This is also the case for *S. pneumoniae* genomes of which 40% (11/28) encode at least one ICE. Among them, seven are derived from clinical isolates known to be resistant to one or multiple antibiotics. All of them carry at least one ICE/dICE suggesting a possible correlation between the presence of ICEs and resistance to antibiotics. Indeed among the families detected, Tn*916* (Roberts and Mullany, 2011), Tn*5252* (Korona-Glowniak et al., 2015), and Tn*1549* (Garnier et al., 2000) are known vectors of antibiotic resistance genes.

### Definition of Different Families of Elements on the Basis of their Conjugative Module

These ICEs were classified into seven distinct families belonging to three superfamilies on the basis of their conjugation modules: (i) Conj_Tn*916*_ and Conj_ICESt3_ belonging to the Conj_Tn*916*_ superfamily, (ii) Conj_*vanG*_, Conj_Tn*5252*_, Conj_Tn*1549*_, and Conj_Tn*GBS2*_ belonging to the Conj_Tn*5252*_ superfamily and the Conj_Tn*GBS1*_ family. The Conj_Tn*916*_ and the Conj_Tn*5252*_ superfamilies of conjugation modules belong respectively to the MPF_FA_ and to the MPF_FATA_ classes of T4SS involved in bacterial conjugation as defined by Guglielmini et al. (2014). No match was found for the Conj_Tn*GBS1*_ family.

### Modular Evolution

The phylogenetic trees obtained for relaxase, CP and VirB4 encoded by elements belonging to the Conj_Tn*916*_ superfamily (**Figure 2**), the Conj_Tn*5252*_ superfamily (**Figure 4**), and the Conj_Tn*GBS1*_ family (**Figure 5**) are highly similar, suggesting that genes exchanges or replacements within the conjugation modules of these families have not occurred or are rare. However, incongruences were found for relaxases, CPs and VirB4 proteins of some elements belonging to ICE*St3* family, suggesting that some gene exchanges or replacements have occurred within the conjugation modules belonging to this family.

Unrelated or very distantly related integrases were found to be encoded by at least some of the ICEs belonging to the same family (except for TnG*BS1* family) and frequently by closely or very related elements (for example in the Tn*5252* family). Furthermore, related site-specific integrases were found in unrelated or distantly related ICEs. Such incongruences are due to multiple exchanges of integration/excision and/or conjugation modules between and/or within ICE families. For most cases, the data do not allow to determine what precisely happened. However, it was previously reported that the last common ancestor of Tn*GBS2* family acquired its DDE transposase from an insertion sequence and that the last common ancestor of Tn*GBS1* family acquired its DDE transposase from an ICE belonging to Tn*GBS2* family (Guérillot et al., 2013). Here, the comparison of phylogenetic trees obtained for CONJ_Tn*5252*_ superfamily, serine integrases and tyrosine integrases clearly shows three independent replacements of the DDE transposases by unrelated integrases. One of these ICEs, *ICE_SagILRI005_rplL*, probably results from: (i) the integration of an ICE belonging to Tn*GBS2* family (encoding a low specificity/site-preferential DDE transposase and a RepA_N protein) into an ICE belonging to Tn*5252* family (encoding a tyrosine integrase specific of *rplL* and another RepA_N protein), and (ii) the loss of the conjugation and replication modules of the Tn*5252*-related element and the deletion of the DDE transposase gene of the Tn*GBS2*-related ICE.

### Integration Specificity of ICEs in Streptococcal Genomes: Impact on Host Fitness and on the Evolution of Elements

In this work, efforts were made to identify the boundaries of the ICEs and therefore to identify the insertion site of each of them. This analysis of integration/excision modules and site specificity is the first one carried for a large array of ICEs encoding their transfer as single-stranded DNA. The ICEs and dICEs of *Streptococci* carry diverse integration/excision modules (75 encoding a tyrosine recombinase, 20 encoding a unique serine recombinase, nine encoding three serine recombinases, and 23 a DDE transposase) and have a large array of integration specificity (low or preferential integration, 18 different site-specific integrations). This work led to the detection of eight new target-genes for streptococcal ICE insertion that have not been identified previously (*ftsK*, *guaA*, *lysS*, *mutT*, *rpmG*, *rpsI*, *traG*, and *ebfC*).

It should be noticed that, among the 131 ICEs/dICEs identified, only nine (restricted to one family) were found to be integrated into the 3′ end of genes encoding tRNAs. This contrasts with the results of the analysis of actinobacterial ICEs (most of these ICEs carry conjugation modules unrelated to the ones of streptococcal ICEs and transfer as double-stranded DNA). Among the 144 actinobacterial ICEs analyzed, 100 were found integrated in the 3′ end of a tRNA gene (Ghinet et al., 2011).

In most streptococcal ICEs, as for almost all other known ICEs, the *attI* site is located in the vicinity of the integrase gene. However, it should also be noticed that for all streptococcal ICEs/dICEs integrated in *rumA*, the *attR* site is located within the serine integrase gene and consequently the integrase gene carried by the excised ICE has a different length and C terminus. *att* sites are found within the genes of their cognate integrase in very few integrative elements, such as the prophage Mx8 from *Myxococcus xanthus* for which the phage attachment site, *attP*, is located within the tyrosine integrase gene (Magrini et al., 1999). Site-specific integration of Mx8 leads to the replacement of the 112-residue C-terminal sequence by a 13-residue C terminus. This modified integrase is less active that the integrase encoded by the excised element. Therefore, it seems probable that the differences between the integrases encoded by the ICEs integrated in *rumA* and the integrases encoded by the excised elements can lead to differences in the function of the two forms of the integrase.

Besides mechanistic constraints leading to integration in conserved palindromic sequences for many tyrosine integrases (Williams, 2002), one would expect that selection criteria for ICE integration in evolution would be (i) to have the least effect on host fitness and, (ii) since many have a large host range (Bellanger et al., 2014), to allow integration into a wide range of strains and species. Numerous ICEs encoding a tyrosine integrase were found to be site-specifically integrated in the conserved 3′ end of essential conserved genes that are isolated or are the last gene of an operon. The insertion does not modify the gene product (tRNA, ribosomal proteins) or leads to very little modification of the 3′ end of the protein (lysyl-tRNA synthetase). Hence, such integrations would have no effect on host fitness and can occur in a large array of species.

All (except one) the streptococcal ICEs belonging to the Tn*916* family detected in this study encode a tyrosine integrase identical or almost identical to the Tn*916* integrase. Analyses of a high number of insertion sites in various hosts showed that Tn*916*, despite having a low specificity of integration, still has a preference for AT rich regions (consensus TTTTTnnnnnnAAAAA; Hosking et al., 1998; Cookson et al., 2011). Furthermore, the analyses of insertion sites after conjugal transfer to *Butyrivibrio proteoclasticus* B316^T^, whose genome has a similar GC percent as the ones of *Streptococci* (39%), showed that only 34% of the 123 analyzed insertions disrupt annotated ORFs even if 90% of this genome is made of ORFs (Cookson et al., 2011). This may be due to lower GC ratio (34.7%) of intergenic regions. Therefore, the AT-rich region preference of Tn*916* probably leads to a null or low impact of most Tn*916* insertion events on host fitness. Since MGEs have generally a lower G+C percent than their host genome (Rocha and Danchin, 2002), this preference could also explain the frequent presence of Tn*916* or Tn*916*-related elements in plasmids or Tn*5252*-related elements (Clewell and Gawron-Burke, 1986; Ayoubi et al., 1991; Ding et al., 2009; Mingoia et al., 2011; Chancey et al., 2015). This putative preference for MGEs would also lead to a lower impact on the fitness of the bacterial host. It was previously shown that a Tn*916* element carried by a Tn*5252*-related element can be transferred alone (Santoro et al., 2010) or as a part of the Tn*5252* element (Ayoubi et al., 1991). Therefore, besides a low impact on host fitness, this A+T rich preference could increase the transfer ability of Tn*916* (either autonomously or by mobilization *in cis*).

Some ICEs integrate in the conserved 5′ end of the first gene of an operon or of an isolated gene that encodes an essential protein (the DNA translocase FtsK that coordinates cell division and chromosome segregation; the nucleoid-associated protein EbfC; the ribosome assembly GTPase RbgA). Importantly, the insertion does not modify the N-terminus of the protein encoded by the target gene. Moreover, for three ICEs integrated in *rbgA* and two ICEs integrated in *ftsK*, the integration does not change the 15–64 bp sequence located upstream from the START codon. However, in the two other ICEs integrated in *rbgA* and in the one integrated in *ebfC*, the sequence upstream from the gene, including its promoter, is completely different, suggesting that the expression of the gene is impacted by the integration of the element. This situation is reminiscent of the integration of the putative satellite prophage SpyCI from *S. pyogenes.* In stationary phase, SpyCI is integrated into the 5′ end of the DNA mismatch repair gene *mutL*, disrupting its expression and that of three other genes located downstream (Nguyen and McShan, 2014). During early exponential growth, SpyCI excises from the bacterial chromosome and replicates as an episome, thus allowing the expression of *mutL* and of downstream genes. Concerning the *ebfC* gene, it is known that in the spirochaete *Borrelia burgdorferi*, it is highly expressed in rapidly growing bacteria but mRNA is undetectable in stationary phase (Jutras et al., 2012). Thus, ICE integration in the promoter of this gene in *S. parasanguinis* FW213 may not alter host fitness at all: expression of *ebfC* gene would not be required in the stationary phase when the element is integrated and excision of the element in the exponential phase restores the expression of this gene.

Tn*GBS1* and Tn*GBS2* are two known elements from *S. agalactiae* encoding DDE transposases that integrate in various intergenic regions located 15 or 16 bp upstream from the 35 box of sigma A promoters (Brochet et al., 2009; Guérillot et al., 2013). In this study, all elements belonging to Tn*GBS1* and Tn*GBS2* families (except three TnG*BS2* that encode serine or tyrosine recombinases) are also integrated in such location. Insertion into intergenic regions is expected to minimize the effects caused by the transposon insertion on host fitness. However, such insertions may interfere with the transcription level of the downstream gene. However, it should be noticed that the insertion of Tn*GBS2* does not seem to affect significantly the transcription level of the gene located downstream from the preferred insertion site.

All insertions of the 29 ICEs encoding serine integrase disrupt genes encoding proteins. A large majority are site-specific. Most of these genes are widespread but are not essential for the strain (*rumA*, *hsdM*, *mutT*). The *rumA* gene encodes a widespread rRNA methyltransferase. In *Escherichia coli*, the deletion of this gene has little effect on growth or on the fidelity of translation, but alters the sensitivity of the ribosomes to fusidic acid and capreomycin (Persaud et al., 2010). The *hdsM* gene encodes the methyltransferase subunit of type I restriction-modification systems (Murray, 2000). The *mutT* gene encodes a Nudix hydrolase that removes oxidized nucleotide precursors so that they cannot be incorporated in DNA during replication (Lu et al., 2001). One element, dICE_*SanC238_traG* is site-specifically integrated into the *traG* gene that encodes the CP of an ICE remnant (not detailed in this report because this remnant is too much decayed) belonging to Tn*1549* family.

The consequences of the integration/excision balance of ICE encoding serine recombinases on the expression of the target genes encoding proteins have never been studied. However, several examples can be cited for prophages or prophage-related elements (Stragier et al., 1989; Kunkel et al., 1990; Rabinovich et al., 2012). Thus, the DNA uptake competence system of the intracellular bacterial pathogen *Listeria monocytogenes* serovar 1/2 was considered non-functional because the competence master activator gene, *comK*, is disrupted by the insertion of the temperate prophage A118 encoding a serine recombinase (Rabinovich et al., 2012). However, the prophage excises not only during the activation of lytic phase but also during intracellular growth, primarily within phagosomes of macrophages, without any production of progeny virions, thus allowing expression of the *comK* gene (Rabinovich et al., 2012). In the same way, ICEs integrated within specific genes and disrupting them may excise when these genes are useful for the host cell (*rumA*, *hsdM*, *mutT*) or for the host ICE (*traG*) to reduce the impact on host fitness and guarantee their maintenance in the cell. Four elements encoding serine integrases are integrated in secondary sites within protein-encoding genes. As for primary integration sites, if the elements are still able to excise, expression of the target gene might not be impacted. The integration of CTn*5*, an ICE belonging to the Tn*1549* family and encoding a serine integrase occurs in an adhesin gene in *Clostridium difficile* 630 (Sebaihia et al., 2006). However, comparison of this genome with that of the derived strain 630Δerm showed that CTn*5* has excised from its original location and has inserted in *rumA* (CD3393) of 630Δerm (van Eijk et al., 2015). This suggests that an ICE integrated in a secondary site is able to excise and reintegrate in its primary site and conversely.

Globally, the impact of the integration upstream from promoters, in the 5′ end of CDSs or within CDSs could be reduced if the ICE excises when the targeted genes are expressed. It was initially thought that ICEs do not replicate autonomously in the cell, although conjugative transfer can be seen as an intercellular replication (Burrus et al., 2002b). Therefore, although the excision could be advantageous for the host, if the cell divides when the ICE is excised, the ICE would be lost in one of the daughter cell. Nevertheless, several recent studies showed or strongly suggested that various single-strand DNA transferring ICEs are capable of extrachromosomal replication in both Gram-positive and Gram-negative bacteria (Carraro et al., 2015 and references therein). In particular, replication was found to be involved in maintenance of Tn*GBS1* and Tn*GBS2* in transconjugants before their integration (Guérillot et al., 2013). These elements encode a protein that carries a repA_N domain and is related to the protein controlling the θ replication of various plasmids and a protein related to ParA, a protein involved in maintenance of some plasmids (Guérillot et al., 2013). We found genes encoding a protein with a RepA_N domain in all ICE belonging to the Tn*5252* family and numerous ICEs belonging to Tn*1549* family. We also found ParA segregation ATPase CDSs in all ICEs belonging to Tn*1549* family and *vanG* family suggesting that all these elements can replicate as episomes. Evidence of intracellular extrachromosomal replication was also recently obtained for ICE*St3* (Carraro et al., 2011) and another element belonging to ICE*St3* family, RD2 (i.e., ICE_*Spy6180_tRNAthr*) of *S. pyogenes* (Sitkiewicz et al., 2011) although these elements do not carry any replication module. Moreover, extrachromosomal replication of ICE*Bs1* from *Bacillus subtilis*, an element belonging to the Tn*916* superfamily (Burrus et al., 2002a) was found to be involved in the stability of the element. This intracellular replication is initiated from the ICE*Bs1 oriT* and required the ICE*Bs1*-encoded relaxase (Lee et al., 2010). At last, all ICEs belonging to the Tn*916* superfamily encode a peculiar relaxase (MOB_T_) related to rolling circle replication initiators involved in maintenance of various plasmids (Guglielmini et al., 2014), suggesting that all these elements are also able to replicate as episomes.

## Conclusion

This study greatly enriches our understanding of the classification and integration sites of ICEs/dICEs in streptococci genomes. In the future, it will be updated and further extended to take into account newly sequenced genomes and to confirm all the trends proposed here. An automated bioinformatics procedure will be developed to keep pace with the constantly growing number of available genomes. Extension to other species of Firmicutes and to the search for IMEs is also envisaged.

## Author Contributions

GG and SP conceived the reference database of signature proteins. GG, SP, NL-B, and M-DD contributed to the conception of the work. CA, CC, GG, NL-B, M-DD, SP, VL, and TL were involved in the acquisition and/or the analysis of the data. NL-B, GG, CC, and SP contribute to the drafting of the manuscript. NL-B and CC elaborated the figures and tables. All authors criticized and finally approved this final version.

## Conflict of Interest Statement

The authors declare that the research was conducted in the absence of any commercial or financial relationships that could be construed as a potential conflict of interest.

## Footnotes

1 http://www.bacterio.net/streptococcus.html

2 http://www.ncbi.nlm.nih.gov/genome/browse/

3 http://central.biomart.org/

4 http://www.ncbi.nlm.nih.gov/Structure/cdd/wrpsb.cgi

5 http://mobyle.pasteur.fr/cgi-bin/portal.py#forms::CONJscan-T4SSscan

6 http://db-mml.sjtu.edu.cn/ICEberg/index.php
